# Design and Implementation of ESP32-Based Edge Computing for Object Detection

**DOI:** 10.3390/s25061656

**Published:** 2025-03-07

**Authors:** Yeong-Hwa Chang, Feng-Chou Wu, Hung-Wei Lin

**Affiliations:** 1 Department of Electrical Engineering, Chang Gung University, Taoyuan City 333, Taiwan; 2 Department of Electrical Engineering, Ming Chi University of Technology, New Taipei City 243, Taiwan

**Keywords:** edge computing, tiny machine learning, ESP32, object detection

## Abstract

This paper explores the application of the ESP32 microcontroller in edge computing, focusing on the design and implementation of an edge server system to evaluate performance improvements achieved by integrating edge and cloud computing. Responding to the growing need to reduce cloud burdens and latency, this research develops an edge server, detailing the ESP32 hardware architecture, software environment, communication protocols, and server framework. A complementary cloud server software framework is also designed to support edge processing. A deep learning model for object recognition is selected, trained, and deployed on the edge server. Performance evaluation metrics, classification time, MQTT (Message Queuing Telemetry Transport) transmission time, and data from various MQTT brokers are used to assess system performance, with particular attention to the impact of image size adjustments. Experimental results demonstrate that the edge server significantly reduces bandwidth usage and latency, effectively alleviating the load on the cloud server. This study discusses the system’s strengths and limitations, interprets experimental findings, and suggests potential improvements and future applications. By integrating AI and IoT, the edge server design and object recognition system demonstrates the benefits of localized edge processing in enhancing efficiency and reducing cloud dependency.

## 1. Introduction

In recent years, the development of Internet of Things (IoT) technology has surged [1,2,3,4], with the integration of artificial intelligence (AI) further accelerating the adoption of IoT applications. A typical IoT framework comprises sensing, transmission, and cloud computing [5,6,7,8]. While the cloud enables vast data processing and storage, uploading all information can strain network bandwidth and raise data security concerns. The rapid growth of IoT and AI is leading to increasing challenges for cloud computing, primarily due to excessive data loads.

Edge computing has emerged as a powerful solution to enhance performance, reduce latency, and improve data security by offloading some processing tasks from the cloud [9,10,11,12]. As IoT devices become more intelligent and resource-intensive, relying solely on cloud computing often results in transmission delays, particularly in time-sensitive applications. By processing data closer to their source, edge computing mitigates these delays and has garnered significant attention in recent research. For instance, studies have demonstrated how resource-constrained edge servers can support smart agriculture, enabling predictive models for water demand and real-time monitoring of plant growth [10]. Additionally, various optimization techniques have been developed to enhance deep learning inference on constrained embedded devices [13]. Processing data at the edge reduces the demand for high-throughput connections, thereby easing the load on cloud resources.

The rise of edge computing is driven by challenges such as cloud data overload, real-time processing needs, bandwidth constraints, and data security concerns. As the volume of data from smart devices continues to grow, edge computing reduces the amount of information transmitted to the cloud, alleviating server strain and improving response times for applications like autonomous driving and security systems. In summary, edge computing lessens dependency on the cloud, enhances real-time responsiveness, optimizes bandwidth usage, and strengthens data security. This makes it crucial to technology for IoT and smart devices, significantly improving overall system performance and user experience. In edge computing, object recognition is particularly effective due to its proximity to the data source, which minimizes bandwidth requirements and reduces latency. This approach has proven beneficial in areas such as smart surveillance, autonomous driving, smart factories, and smart homes. For example, edge computing has enhanced contamination detection in mushroom logs [14], enabled real-time object detection for estimating bottom-trawl catch volumes [15], and simulated optical sensor networks to monitor athlete performance [16]. In addition, a distributed deep reinforcement learning-based quantization scheme was proposed to optimize training time and quantization error in federated learning enabled vehicle edge computing, reducing latency and handling time-varying channel conditions effectively [17].

Object detection in edge computing enables real-time tasks such as tracking, classification, and segmentation, supporting applications in smart cities, healthcare, autonomous vehicles, and industrial automation. Integrating edge computing with lightweight deep learning techniques helps overcome challenges in deploying high-performance models in resource-constrained environments, including limited resources, the latency-accuracy trade-off, energy efficiency, and model deployment. In [18], the study found that the YOLO-v4-LSAM model is effective for real-time object detection in mining environments. Similarly, in [19], EL-YOLO was identified as an efficient solution for real-time small object detection in low-altitude aerial images, balancing accuracy, speed, and computational efficiency, making it suitable for resource-limited edge devices. For UAV applications, YOLO-based object detection enhances landing by improving real-time recognition, obstacle avoidance, and decision-making [20]. In fire detection, YOLO models integrated with cloud–edge collaboration and transfer learning improve accuracy, reduce false alarms, and dynamically adapt to real-time fire prevention scenarios [21]. In [22], a vehicular detection architecture was proposed to detect abandoned objects, leveraging task-based AI technology for large-scale road maintenance in mobile computing environments. Additionally, in [23], object detection methods were explored to accurately identify fire and smoke sources in large-scale settings, demonstrating their effectiveness across diverse real-world applications.

Data privacy in edge computing is crucial due to the sensitive nature of processed data, the distributed structure of edge systems, and the demand for real-time processing. Ensuring security, regulatory compliance, and user trust requires advanced cryptographic techniques and user-centric designs. Recent research has introduced several innovative solutions. An efficient attribute-based searchable encryption scheme enables secure keyword searches and outsourced decryption for lightweight devices [24]. A privacy protection mechanism effectively balances privacy, data utility, and real-time performance in mobile crowdsourcing systems [25]. In edge camera applications, a direct edge-to-edge transfer learning approach enhances accuracy, reduces transmission costs, and strengthens privacy [26]. For vehicular networks, a flexible data-sharing framework for VANETs allows fine-grained erasure and secure communication, improving both efficiency and privacy [27]. Optimized homomorphic encryption and faster protocols in Panther accelerate secure two-party neural network inference with significant speed improvements [28]. A privacy-preserving protocol ensures secure, verifiable, and efficient parallel matrix determinant computation for IoT devices [29]. Furthermore, a practical solution balances privacy and utility in spatial crowdsourcing, making it applicable to real-world scenarios [30].

Edge–cloud collaboration has shown great potential to optimize IoT system performance by distributing tasks between edge devices and cloud servers. This approach effectively addresses challenges such as latency reduction, resource efficiency, and real-time responsiveness [31,32,33,34]. Recent advancements in edge–cloud collaboration have focused on task scheduling, communication efficiency, and privacy-preserving mechanisms, significantly enhancing IoT systems. Dynamic offloading and task scheduling strategies have been proposed to explore energy-efficient task management and workflow optimization [35,36]. Techniques like dynamic voltage scaling and reinforcement learning reduce energy consumption, latency, and costs, enabling efficient hybrid systems tailored for real-time IoT applications. Communication efficiency has also emerged as a key focus area. The studies [37,38] highlight advancements in bandwidth optimization and secure transmission through techniques such as advanced compression, clustering mechanisms, and searchable encryption, ensuring effective communication in bandwidth-constrained environments. Practical applications have demonstrated the feasibility of edge–cloud systems for latency-sensitive and time-critical tasks. For instance, industrial and vehicular IoT implementations [39,40] leverage time-sensitive networking protocols and dynamic resource allocation to deliver scalable solutions for real-time fault detection, machine control, and vehicular network management. Further optimization techniques, including reinforcement learning and asynchronous collaboration [41,42], dynamically adjust resource allocation to improve robustness, efficiency, and adaptability to workload variations. However, while these studies provide robust frameworks, they lack detailed exploration of resource-constrained hardware like ESP32-CAM, which is the focus of this work.

This study aims to validate the benefits of using a single-chip microprocessor, specifically the ESP32-CAM, as an edge server. Although edge servers are typically less powerful than cloud servers, they can cost-effectively enhance cloud performance by offloading trivial tasks, thereby reducing the burden on expensive cloud infrastructure [43,44,45]. This research integrates IoT concepts by enabling data collectors to function as edge servers, performing local pre-processing before uploading data to the cloud. This approach reduces network resource usage and cloud computing demands, ultimately optimizing IoT system performance. Recently, there has been growing interest in deploying on-device machine learning applications using ESP32 series microcontrollers [45,46,47,48]. For instance, a monitoring system that combines the ESP32-CAM and Raspberry Pi with YOLOv8 has been developed to detect and classify objects, such as humans and animals, in the field [46]. Additionally, a dynamic AI-IoT architecture has been proposed, utilizing 5G as a bridge between edge and low-powered IoT devices, enabling the deployment of TinyML models in various scenarios [47]. Moreover, an ESP32-based real-time distance estimation system using various training algorithms has also been implemented [48].

With the advancement of IoT and edge computing, there has been growing interest in Tiny Machine Learning (TinyML) [49], which enables machine learning on single-chip microprocessors. To accommodate constrained devices, models must be simplified, and parameter counts minimized. This challenge has led to the development of frameworks such as Google TensorFlow Lite for Microcontrollers, which facilitates machine learning on microcontroller units (MCUs). Several platforms support TinyML development, including Edge Impulse [50], Fraunhofer IMS AIfES [51], AITS cAInvas [52], and SensiML [53]. Among these, Edge Impulse stands out for its user-friendly interface, which simplifies building and deploying machine learning models on edge devices. As IoT devices proliferate, the demand for real-time data processing has grown, particularly in applications like object recognition. Traditional cloud-based systems struggle to meet the low-latency requirements of these applications, especially when network congestion or bandwidth limitations are present. Edge computing offers a solution by processing data locally, but many edge devices have limited computational resources, making them unsuitable for more complex tasks such as deep learning-based object detection. To address this challenge, this study explores a hybrid edge–cloud architecture, where the edge device performs initial processing and the cloud handles more computationally intensive tasks, such as object verification. By leveraging the ESP32-CAM on the edge and YOLOv8 in the cloud, we propose a system that optimizes both latency and accuracy. This work provides insights into the integration of edge and cloud computing in real-time object recognition systems, with a focus on efficiency and scalability in resource-constrained environments.

While previous studies have investigated edge computing for object detection, they have primarily focused on more powerful embedded devices. The specific performance of highly constrained hardware like the ESP32-CAM in edge computing scenarios has not been extensively explored. Existing research has shown that edge computing reduces latency, but few studies have systematically analyzed the trade-offs between edge-side pre-processing and cloud-based verification. This study investigates how a hybrid edge–cloud approach can optimize latency, accuracy, and resource usage in real-time object recognition systems. Leveraging the ESP32-CAM’s local processing capabilities while selectively offloading complex tasks to the cloud, the proposed system offers a cost-effective and efficient solution for resource-constrained IoT devices. By integrating edge and cloud computing, the system reduces latency and bandwidth usage while maintaining high recognition accuracy through cloud verification. This study also provides empirical data comparing different MQTT broker configurations and image size adjustments, highlighting how these factors impact transmission time and recognition performance. Our experiments demonstrate that this hybrid approach is particularly advantageous in scenarios with intermittent connectivity or constrained resources. Edge pre-processing triggers immediate actions, while cloud processing verifies results when needed. The primary objective of this study is to evaluate the integration of edge computing and cloud processing for real-time object recognition using the ESP32-CAM. The focus is on assessing the trade-offs between latency, accuracy, and resource usage in hybrid systems while providing practical recommendations for optimizing IoT deployments. The contributions of this paper can be summarized as follows:

1. Develop and evaluate a hybrid system combining ESP32-CAM edge computing with cloud processing for real-time object recognition, balancing latency, accuracy, and resource efficiency.
2. Conduct a detailed analysis of the ESP32-CAM as a low-cost, resource-constrained edge device, evaluating its feasibility and limitations in IoT applications.
3. Investigate the effect of local and remote MQTT broker configurations on system latency, providing optimization strategies for communication in IoT networks.
4. Explore trade-offs between image size, latency, and MQTT broker selection to provide insights for optimizing IoT system configurations.
5. Propose strategies to address connectivity challenges, such as offline modes and lightweight edge-side models, to improve adaptability in real-world IoT environments.
6. Demonstrate the broader implications of integrating low-cost edge devices with cloud services, offering insights for future IoT system designs in smart cities, smart buildings, and remote monitoring applications.

## 2. Materials and Methods

### 2.1. Edge Computing Architecture

The system architecture is designed to enable efficient real-time object detection on a single-chip ESP32 microprocessor (TaiwanSensor, Tainan, Taiwan). The ESP32-CAM is a low-cost, compact development board that combines the ESP32 microcontroller with an onboard camera module, making it suitable for IoT applications involving image and video capture. It features a built-in OV2640 camera (TaiwanSensor, Tainan, Taiwan) module that supports image resolutions of up to 1600 × 1200 pixels, which contributes to its popularity in cost-effective edge computing solutions. In this study, the ESP32-CAM was utilized to capture images with its onboard camera. After capturing the images, the visuals were displayed on a 1.8-inch 128 × 160 TFT-LCD ST7735S (TaiwanSensor, Tainan, Taiwan), while image recognition was performed simultaneously. The recognition results were output to the TFT-LCD ST7735S, and the detailed image data were transmitted via UART to the Arduino IDE [54].

**Figure 1:**
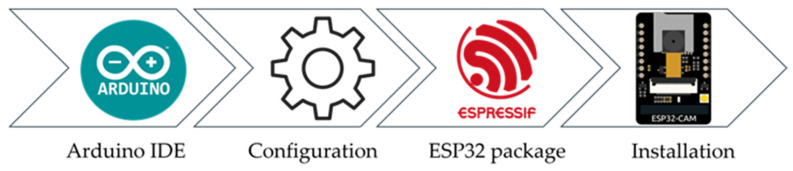
Installation process of ESP32-CAM in Arduino IDE.

**Figure 2:**
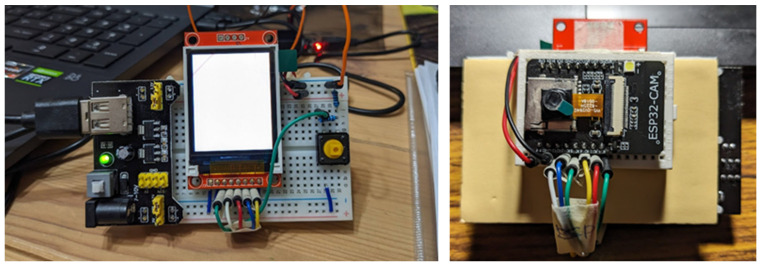
ESP32-CAM module: 1.8-inch LCD (**left**), onboard camera (**right**).

The Arduino IDE provides a user-friendly graphical environment that simplifies development, making it convenient to use. The installation process for the ESP32 in the Arduino IDE is outlined in Figure 1. After downloading and installing the IDE from the official website, users can configure necessary settings, such as Preferences and Additional Boards Manager URLs. These configurations enable support for various hardware platforms, allowing the IDE to be customized for different projects. Next, the ESP32 package by Espressif Systems can be found and installed through the Boards Manager. Once installed, the AI Thinker ESP32-CAM can be selected from the Board menu under Tools to ensure proper configuration for uploading and communicating with the ESP32-CAM hardware. Figure 2 shows the ESP32-CAM module used in this paper for implementing edge computing.

### 2.2. Communication Protocol

In this study, the built-in Wi-Fi module of the ESP32-CAM was utilized to connect to the Internet and transmit images and text messages to a laptop serving as a cloud server via the Message Queuing Telemetry Transport (MQTT) protocol. MQTT is widely adopted in IoT systems due to its lightweight design and support for topic-based publish–subscribe architectures [55,56,57]. In the MQTT framework, multiple IoT devices connect to a server known as a broker, which facilitates efficient data transmission and reduces network overhead. The use cases of MQTT in IoT are diverse, including applications in smart cities, smart farms, and smart manufacturing [58,59,60,61].

**Figure 3:**
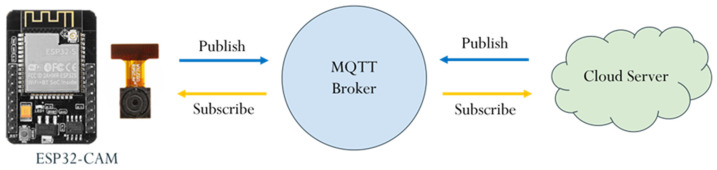
MQTT communication in the edge–cloud environment.

This study employs the MQTT protocol for communication with the cloud server. The edge device, ESP32-CAM, publishes images or recognition results, which are sent to the cloud laptop via the MQTT broker. Upon receiving the message, the laptop publishes the results back to the ESP32-CAM. Both the edge device and the cloud server function as publishers and subscribers, as illustrated in Figure 3.

In Figure 3, the MQTT broker serves as a central server that manages message routing between clients. It receives messages from publishers and forwards them to the appropriate subscribers based on the specified topics, ensuring efficient and reliable communication between devices. A topic is a string used by the broker to filter and route messages to the correct clients, acting as an addressing mechanism. Publishers send messages to specific topics, while subscribers receive messages by subscribing to those relevant topics. Publishing refers to the act of sending a message to a specific topic. A device that generates data (the publisher) transmits its message to the MQTT broker with a defined topic. The broker then forwards this message to any clients subscribed to that topic. Subscribing involves registering for a specific topic to receive messages. Clients can subscribe to one or more topics, and whenever a message is published on any of those topics, the broker delivers it to the respective subscribers.

### 2.3. Edge Server Design

**Figure 4:**
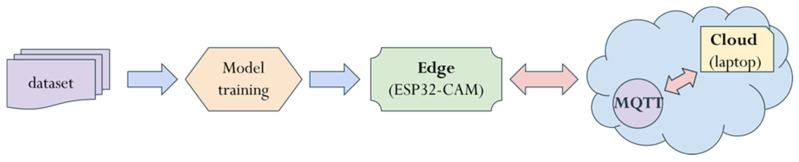
Overall software framework.

This study investigates the benefits of using edge devices to support cloud computing. The selection of the edge device focused on low cost and low power consumption, with the aim of effectively reducing the load on high-cost, high-power cloud servers. As a result, the single-chip processor ESP32-CAM was chosen as the edge device, while a laptop served as the cloud server to facilitate operation and analyze differences in latency between the edge and cloud. Communication between the edge and cloud was established using the lightweight MQTT protocol, which simplifies communication and reduces system complexity. The ESP32-CAM at the edge monitored video, and when a person was detected, it sent the corresponding message or image to the cloud via MQTT. The cloud processed the data and sent an action command back to the edge device. By pre-processing data on the edge, the system could minimize the cloud’s continuous video monitoring load and enable quicker responses, such as sending alerts, turning on lights, or recording video. The overall software framework is illustrated in Figure 4.

**Figure 5:**
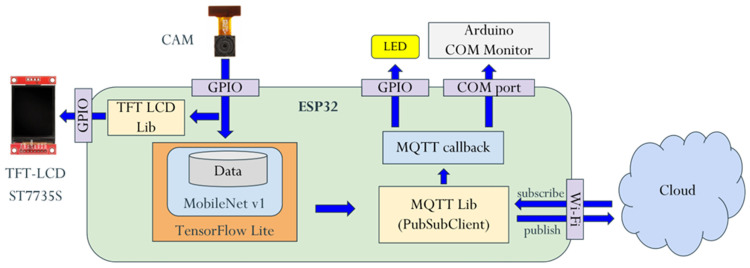
The software framework for the ESP32-CAM edge device.

The software framework for the ESP32-CAM edge device is illustrated in Figure 5. The camera module captures images at resolutions of 96 × 96 RGB565 (or 640 × 480 JPEG, 320 × 240 JPEG), which are then sent to the recognition module. Here, the image is converted to 96 × 96 RGB888 for processing by a MobileNet model running on TensorFlow Lite. MobileNet is a lightweight deep learning model specifically designed for efficient image classification on resource-constrained devices [62,63,64]. In this study, MobileNet is employed as the backbone of a simplified image recognition system, where its classification capabilities are utilized to distinguish between person and non-person categories. Its architecture reduces computational complexity, enabling fast, real-time recognition tasks while consuming minimal power. TensorFlow Lite is optimized for edge devices, allowing for the deployment of the MobileNet model in a highly efficient format, which facilitates on-device inference with reduced memory usage and improved speed. Simultaneously, the image is displayed on an external TFT LCD screen, also converted to 96 × 96 RGB565. Once the MobileNet model processes the image and classifies the objects, the recognition results are transmitted to the MQTT broker using the MQTT library. When the MQTT subscriber receives the message, it processes the data via a callback function, triggering actions such as lighting up an LED or sending logs to the Arduino Serial Monitor for performance analysis. If edge-side pre-recognition and pre-processing are enabled, the action is executed immediately after recognition on the edge device. In some cases, the action may be verified by the cloud and adjusted based on feedback sent via MQTT. In addition, based on the ESP32-CAM datasheet, the device’s power consumption varies depending on its operational state, ranging from less than 1 mA in deep-sleep mode to over 200 mA during peak activity, such as image capture and Wi-Fi transmission.

### 2.4. Cloud Server Design

**Figure 6:**
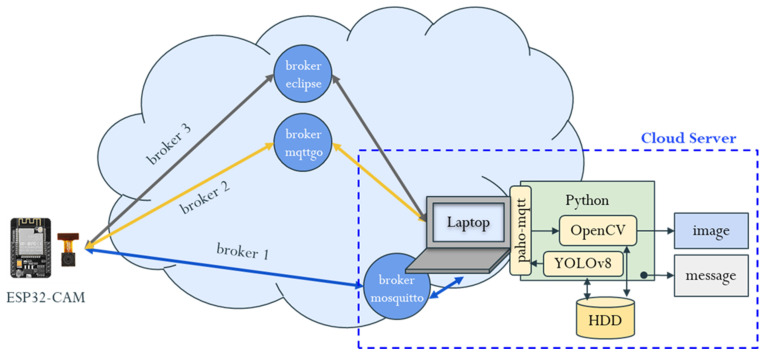
Cloud server software framework.

In this study, a laptop running Windows 11 served as the cloud server. The cloud-side program was developed using Python 3.12.4, with several key packages installed, including the MQTT package, OpenCV, and YOLOv8. The design of the cloud server software framework is illustrated in Figure 6. The emergence of YOLO has improved the speed and efficiency of object detection, enabling real-time processing. This breakthrough has made YOLO highly versatile, with applications across a wide range of fields [65,66,67]. The ESP32-CAM, functioning as the edge device, publishes images or messages to an MQTT broker over the network. The laptop, serving as the cloud server, subscribes to the corresponding MQTT topics to receive these images or messages. To compare edge–cloud latency and evaluate the benefits of edge computing across varying network distances, three different MQTT brokers were selected: Mosquitto, MQTTGO, and Eclipse Projects.

Mosquitto is an open source message broker that implements the MQTT protocol, designed for lightweight, low-latency communication in IoT networks. In this setup, Mosquitto runs as a locally self-hosted broker on a server within the same local area network as the ESP32-CAM. This configuration ensures the lowest possible latency, as data do not need to travel over the internet. MQTTGO is a cloud-hosted broker that, in this case, operates on a server geographically close to the local network where the ESP32-CAM is deployed. This setup leverages cloud infrastructure to provide higher scalability and uptime while still maintaining relatively low latency due to the server’s proximity. Eclipse Projects is a globally recognized open source platform that offers free MQTT brokers for public use. In this study, the Eclipse broker is hosted remotely, meaning the ESP32-CAM must transmit data over the internet to a server located in a different region or country, which introduces higher latency due to the greater distance.

On the cloud laptop, the Python program utilizes the paho-mqtt library to send and receive messages. Upon receiving an image from the edge device, the program employs OpenCV to save the image to the hard drive and display it for testing and verification. OpenCV also supports object recognition; however, due to its limitations in human recognition performance, this study opted for YOLOv8 for cloud-based re-verification. YOLOv8 performs object detection on the saved images, drawing bounding boxes and labeling detected objects. The labeled images are then saved to the hard drive for review and confirmation. As the latest iteration in the YOLO series, YOLOv8 represents a significant advancement in object detection models [68,69,70,71].

### 2.5. Model Training and Deployment

**Figure 7:**
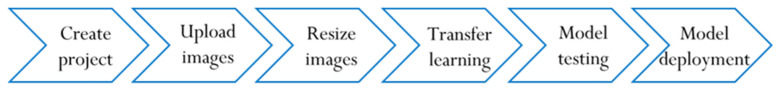
Entire process from data collection to model deployment.

In this study, the person recognition model was retrained using the Edge Impulse platform, a web-based tool designed to simplify the processes of data collection, labeling, and model training for embedded devices. Edge Impulse also allows seamless deployment by exporting trained models as Arduino libraries, which include example codes for direct integration into edge devices. Using the Arduino IDE, the trained model was installed and uploaded onto the ESP32-CAM for real-time inference and testing. The entire process, from data collection to model deployment, is illustrated in Figure 7.

We selected MobileNet as the backbone model for edge-side object detection due to its lightweight architecture and efficiency in resource-constrained environments. MobileNet utilizes depthwise separable convolutions, which significantly reduce computational complexity and memory usage while maintaining high classification accuracy. Given the limited processing power and memory of the ESP32-CAM, deploying more complex deep learning models would be infeasible. MobileNet provides an optimal balance between accuracy, speed, and efficiency, making it well-suited for real-time inference on low-power IoT devices.

The training dataset was sourced from the ImageNet ILSVRC 2017 library [42], a large-scale image database commonly used in computer vision research. From this dataset, we selected 4643 images labeled as “person” and 4500 images labeled as “non-person” to train the classification model. Model training was conducted using Edge Impulse’s user-friendly web interface, which supports three project types: Motion (gesture recognition), Images (object detection), and Audio (sound classification). Since this study focuses on object detection, we selected the Images project type. The dataset was uploaded to Edge Impulse, and images were manually labeled as either “person” or “non-person” before training. To ensure optimal training and compatibility with the ESP32-CAM, images were resized to 96 × 96 pixels, and the Fit longest mode was applied to retain key visual features.

We employed Transfer Learning, selecting a pre-trained MobileNet model, which eliminated the need to train the network from scratch. This method enhances performance while reducing training time and computational overhead. In Transfer Learning, two key steps were performed: Neural Network Settings—Training parameters such as the number of epochs and learning rate were adjusted to improve model performance. Neural Network Architecture Selection—We compared MobileNet v1 and MobileNet v2, ultimately selecting MobileNet v1 due to its lower computational requirements, making it more suitable for the ESP32-CAM. Before deployment, we tested the trained model using Edge Impulse’s Model Testing feature, reserving 20% of the dataset (1829 images) for validation to assess accuracy and robustness.

To deploy the model, we exported it as an Arduino library compatible with TensorFlow Lite for Microcontrollers. The model was then installed and uploaded onto the ESP32-CAM using the Arduino IDE. During execution, the ESP32-CAM captures images, processes them locally using the trained MobileNet model, and determines whether a “person” is present. The recognition results are displayed on an external TFT-LCD screen and transmitted via MQTT for cloud verification. This hybrid edge–cloud approach enhances performance by leveraging edge-side MobileNet inference for low-latency decisions, while cloud-based YOLOv8 verification ensures higher accuracy and reduces unnecessary data transmission. This setup significantly reduces bandwidth consumption while maintaining reliable detection.

### 2.6. Performance Evaluation Metrics

The experiments in this study consist of three main components: the edge-side ESP32-CAM, a cloud-based laptop, and the MQTT broker for data and image transmission. Each cycle performed by the ESP32-CAM involves the following steps:

1. Image Capture: The image format can be set to RGB565 or JPEG, with various resolutions, including 96 × 96, 160 × 120, 240 × 240, 320 × 240, and 640 × 480. Higher resolutions require more time and system resources.
2. Image Conversion: The captured image is converted to RGB888 and resized to 96 × 96 to match the input requirements of the MobileNet v1 model used in this study.
3. Object Recognition: After the image is processed by the object recognition model, confidence scores for each label are generated. In this study, any score of 0.5 or higher for the “person” label is considered a positive recognition.
4. MQTT Publish: ESP32-CAM publishes the image or a message to the MQTT broker.
5. MQTT Subscribe: The ESP32-CAM receives messages published by the cloud.

Initially, a resolution of 96 × 96 pixels with the RGB565 format was used to optimize processing speed on the ESP32-CAM. However, this low resolution made recognition challenging for both the human observer and the model. To enhance recognition capabilities, the resolution of the images sent to the cloud increased to 640 × 480 pixels. Given that BMP images at this resolution are too large (640 × 480 × 3 = 900 KB), the image format was switched to JPEG to reduce file size while maintaining image quality.

## 3. Results

### 3.1. Test Platform and Configuration

**Figure 8:**
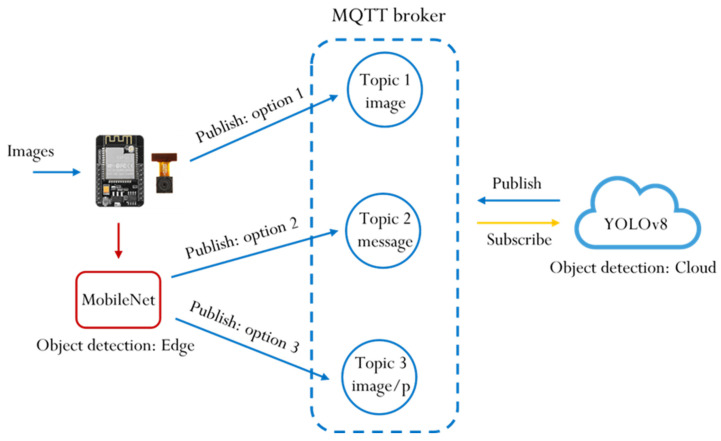
The experimental setup for the image capture and recognition.

This study employs the ESP32-CAM for image capture and person recognition, as shown in Figure 8.

In the experiment, the ESP32-CAM was directed at a laptop screen that switched every 0.5 s between pre-selected images of scenes with and without people. The program in the ESP32-CAM alternated between three options during the experiment:

Option 1: Only send the image to the MQTT broker without performing recognition.

Option 2: Perform recognition and only send a message to the MQTT broker.

Option 3: When a person is detected, send the image to the MQTT broker.

These options are executed sequentially across three different MQTT brokers:

Broker 1: Mosquitto, a local MQTT broker set up on the laptop.

Broker 2: MQTTGO, a locally based MQTT broker.

Broker 3: Eclipse Projects, an international MQTT broker.

There are a total of nine combinations. Each combination runs 100 consecutive iterations, resulting in 900 iterations to complete one experiment.

In the cloud processing phase, four Python programs are executed. One program utilizes OpenCV to read and display images from a designated folder, switching images every 0.5 s. The remaining three programs manage responses for each of the MQTT brokers. For Option 2, where only a message indicating either “person” or “non-person” is sent, the cloud responds with a simple “got it” acknowledgment. For Options 1 and 3, which involve image transmission, the process begins by saving the received image. OpenCV then opens the image file, and YOLOv8 performs object detection, marking bounding boxes around the detected objects. The detection results are saved and displayed on the screen. Finally, the person-detection outcomes from YOLOv8 are published as a message to the MQTT topic for the edge device to receive.

### 3.2. Testing Images

**Figure 9:**
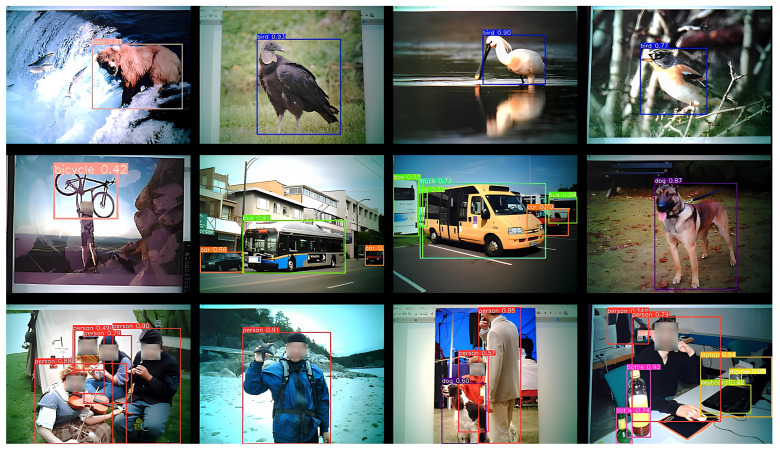
Samples of testing images.

This study conducts experiments using an ESP32-CAM to capture images of a laptop screen displaying content from the ImageNet ILSVRC 2017 dataset. This dataset contains 20,121 images classified into two categories: “person” and “non-person”. A specific proportion of these images is mixed to create a video where the image changes every 0.5 s, resulting in a total runtime of 10 min and encompassing 1200 images. The default scenario features a composition of 30% “person” images and 70% “non-person” images. Figure 9 illustrates a test image set captured by the ESP32-CAM, showcasing the rotating images on the laptop screen. After being transmitted to the laptop via MQTT, these images undergo processing and recognition using the YOLOv8 model. Each image is numbered according to the loop sequence corresponding to each experimental option.

### 3.3. Performance Comparison and Analysis

**Figure 10:**
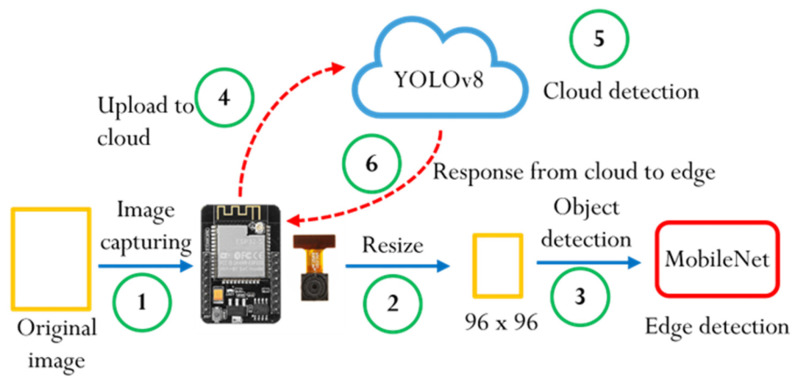
Complete object detection process in the edge–cloud system.

The process in this study begins with the ESP32-CAM capturing an image, followed by a series of transformations and analyses, both on the edge device and in the cloud, shown as Figure 10. The explanations are described below:

1. Image capture: The ESP32-CAM captures an original image, which can be in a variety of formats such as RGB565 or JPEG, at resolutions such as 96 × 96 or 640 × 480.
2. Image resizing: The captured images are resized to 96 × 96 for local processing on the ESP32-CAM, ensuring that the images are suitable for MobileNet processing.
3. Object detection (Edge): Using the resized image, the ESP32-CAM performs object detection via the MobileNet model. This lightweight model is used for edge devices to recognize whether a “person” is present.
4. Upload to cloud: Once the local detection is completed, the original high-resolution image (e.g., 640 × 480 JPEG) is uploaded to the cloud server via the MQTT protocol for further analysis.
5. Cloud detection (Cloud): In the cloud, the high-resolution image is processed using the YOLOv8 model, which provides more detailed object recognition. YOLOv8 could identify multiple objects in the image with a high degree of accuracy.
6. Cloud-to-Edge response: After YOLOv8 processes the image and identifies objects, the cloud server sends the detection results back to the ESP32-CAM via MQTT.

**Figure 11:**
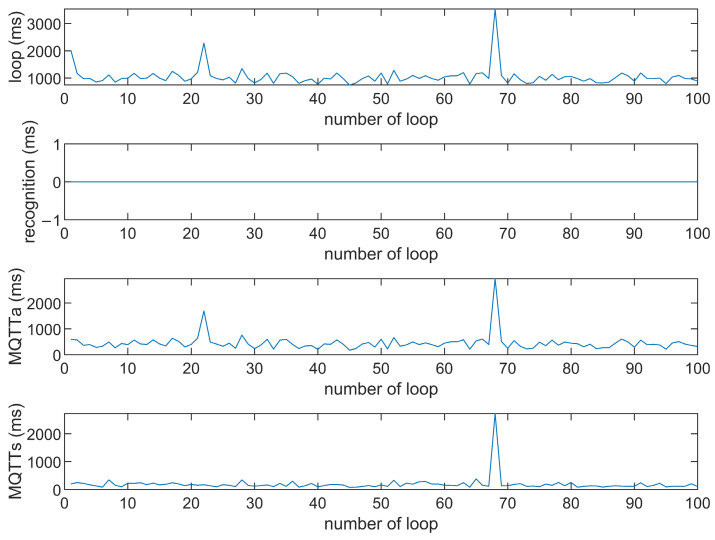
Response time of the Mosquitto broker (Option 1).

**Figure 12:**
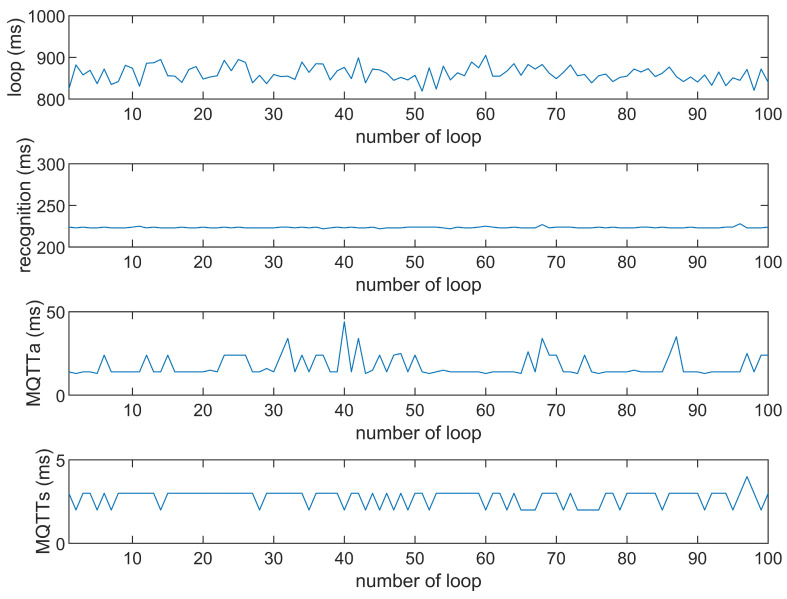
Response time of the Mosquitto broker (Option 2).

**Figure 13:**
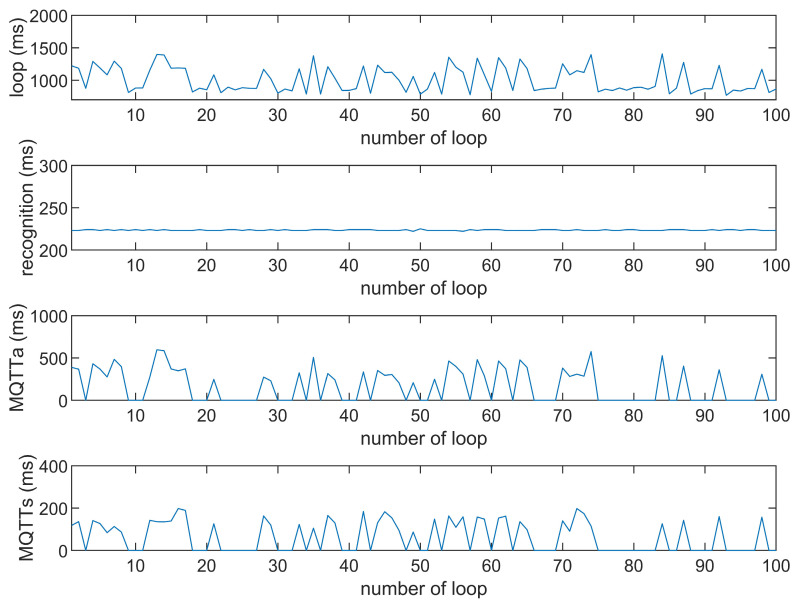
Response time of the Mosquitto broker (Option 3).

**Figure 14:**
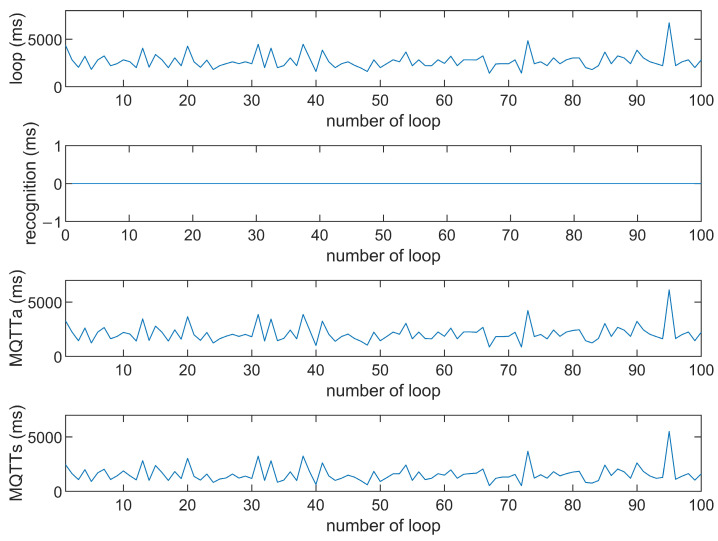
Response time of the MQTTGO broker (Option 1).

**Figure 15:**
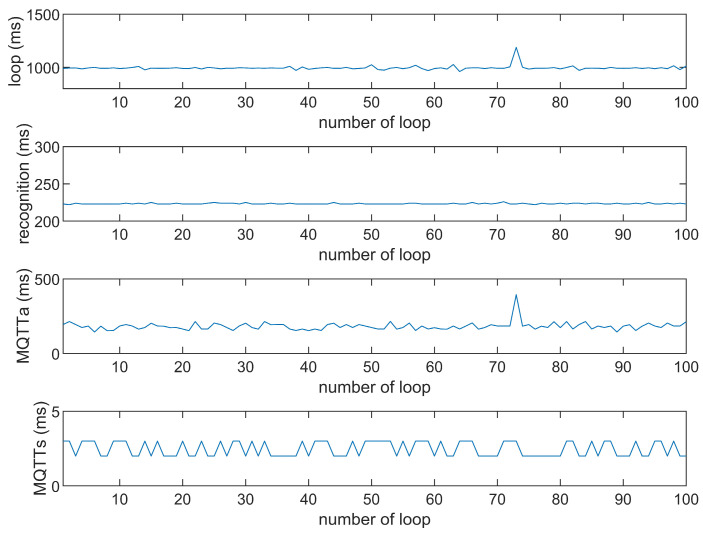
Response time of the MQTTGO broker (Option 2).

**Figure 16:**
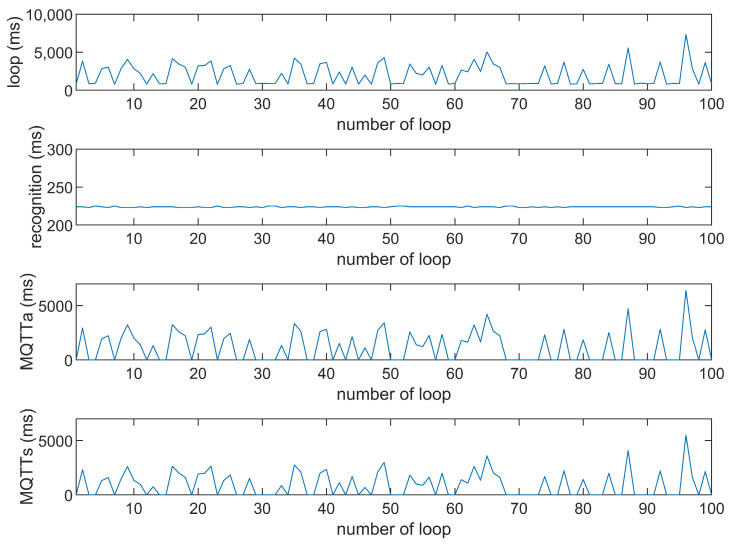
Response time of the MQTTGO broker (Option 3).

**Figure 17:**
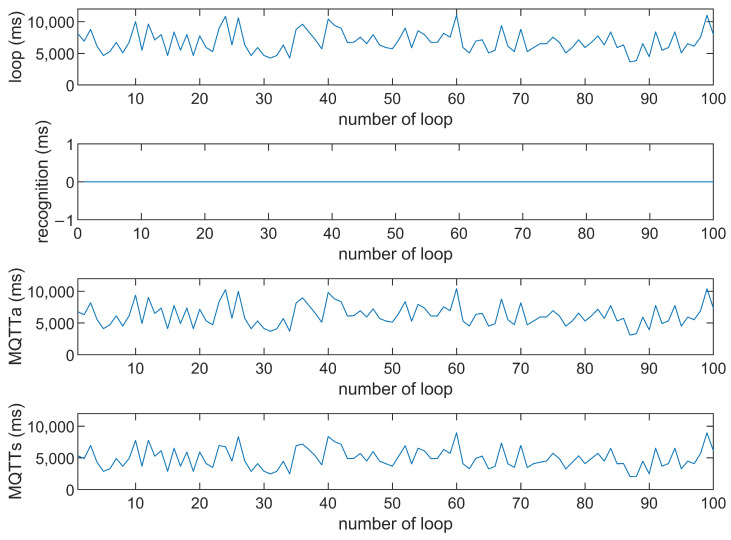
Response of the Eclipse broker (Option 1).

**Figure 18:**
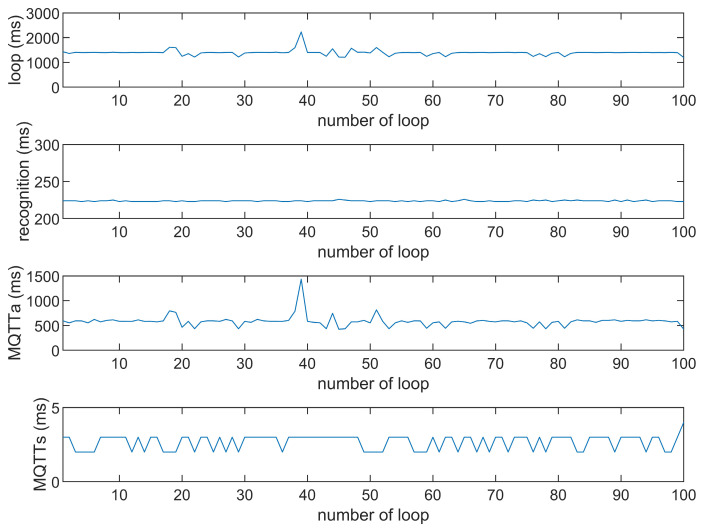
Response time of the Eclipse broker (Option 2).

**Figure 19:**
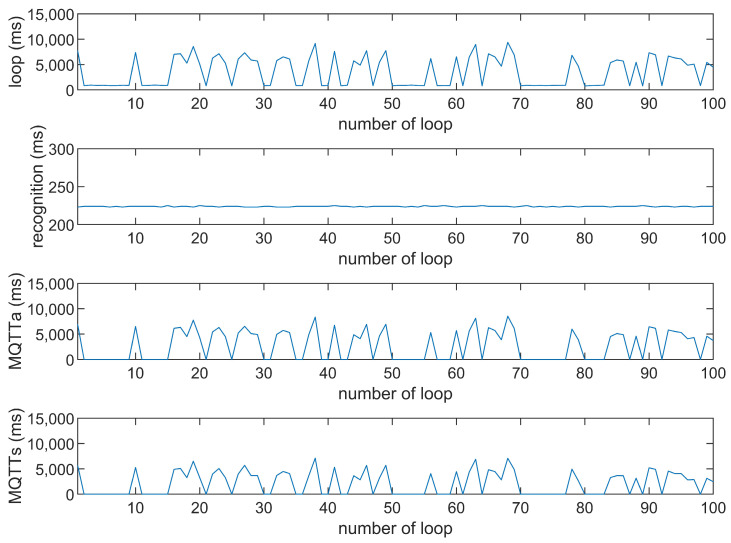
Response time of the Eclipse broker (Option 3).

This study compares the average execution times of three options, Option 1: “send image for all”, Option 2: “send message for all”, and Option 3: “send image when person detected”. The comparison is made across three MQTT brokers at different distances: cloud laptop local, domestic MQTTGO, and international Eclipse. Each option is tested over 100 iterations. As shown in Table 1 and Figure 11, Figure 12, Figure 13, Figure 14, Figure 15, Figure 16, Figure 17, Figure 18 and Figure 19, the experimental data reveal that, when sending images (640 × 480 JPEG files) to a domestic broker, the average time per full iteration is 1655 ms longer (2.58 times) compared to sending to a local MQTT broker sharing a Wi-Fi hotspot. When connecting to an international broker, the average time increases by 5815 ms (6.55 times). These data illustrate the impact of network latency, which varies with the distance to the server. When network latency is significant, reducing the size of transmitted data can effectively minimize their impact. For example, the total time to send messages to the Eclipse server is only 20.34% of the time required to send images, indicating that reducing the amount of transmitted data can effectively mitigate the effects of latency.

In Figure 11, Figure 12, Figure 13, Figure 14, Figure 15, Figure 16, Figure 17, Figure 18 and Figure 19, loop time refers to the total time required to complete the entire process—from the ESP32-CAM capturing an image, through local processing and cloud-based recognition, to the point when the cloud server sends the results back to the ESP32 edge device. MQTTa represents the total time for MQTT data transmission, while MQTTs specifically refers to the portion of MQTT communication time constrained to image transmission. Additionally, recognition time is the time taken to perform object recognition.

Option 1 represents an extreme setup where all images are sent to the cloud for recognition, with no edge-side processing. In contrast, Option 2 handles all recognition tasks on the edge device, transmitting only messages to the cloud. This study adopts a balanced approach with Option 3, where initial recognition occurs at the edge to enhance real-time performance, while the cloud verifies results to improve accuracy. Additionally, recognition results for non-person images are omitted in Option 3 to save time, making it ideal for scenarios with infrequent human presence. Table 1 compares the average time taken for the entire system to complete a full cycle from the ESP32-CAM transmitting data to the laptop processing and returning results. In Table 1 and all other tables, “Mean” represents the average value, and “SD” stands for the standard deviation. To assess the impact of network latency, MQTT transmission times are analyzed separately. Table 2 details the time for edge publishing, cloud processing, and cloud-to-edge result transmission.

In Option 1, where images are sent without recognition, Table 2 shows that the MQTTGO broker introduces an additional 1663 ms compared to the local Mosquitto broker, while the Eclipse broker adds 5816 ms more than the local broker. These delays are significant and would be intolerable for systems with strict timing requirements. In contrast, in Option 2, where only messages are sent, MQTTGO adds 165 ms more than the Local broker, and Eclipse adds 567 ms more. These differences are much less significant compared to Option 1. Similarly, the MQTT latency for the international broker (Eclipse) is significantly higher than that of the other two brokers across all options. This increased latency impacts overall system performance, especially in configurations where timely communication is critical. The consistently higher delays introduced by the international broker make it less suitable for applications requiring real-time responsiveness, while the local and regional brokers (Mosquitto and MQTTGO) offer more manageable latency.

Figure 11, Figure 12, Figure 13, Figure 14, Figure 15, Figure 16, Figure 17, Figure 18 and Figure 19 illustrate the experimental results obtained using different MQTT brokers and image sizes. The subsequent analyses provide insights to help readers interpret these results effectively.

1. Figure 11 (Broker Local, Option 1) shows the average loop time when images are sent without recognition. The relatively low latency observed in this case is primarily due to the minimal processing required by the edge server, as the cloud does not perform object detection. However, network factors, such as bandwidth and transmission distance, still contribute to the total latency.
2. In contrast, Figure 12 (Broker MQTTGO, Option 1) illustrates an increase in loop time when using a domestic MQTT broker. This performance change can be attributed to the added network latency introduced by the geographically distant server. The performance degradation in this setup is more significant compared to the local broker due to the increased data transmission distance.
3. Figure 13, Figure 14, Figure 15 and Figure 16 (Options 2 and 3, across all brokers) highlight the impact of sending only messages versus images. As shown in Figure 13, when only messages are sent, the latency is significantly lower. This occurs because smaller message payloads require less transmission time and bandwidth, reducing network congestion.

We explain these trends by examining the interaction between the data size, the broker’s location, and the network conditions. Larger image sizes, for example, increase transmission time and bandwidth usage, leading to higher latency. Similarly, the use of more distant MQTT brokers exacerbates network delays.

Furthermore, the impact of loop time for the remote broker (Eclipse) at different times is analyzed, as shown in Table 3. The results indicate some variation in loop times, but the differences between night, morning, and evening are not significantly large. In Option 1, the mean loop time remains consistently high across all times. The large standard deviation suggests significant variability. In Option 2, the loop time is much lower compared to the other options, with a small SD. In Option 3, the loop time is about half of the loop time in Option 1. However, the very high SD indicates a high level of variability relative to the mean, suggesting inconsistent performance.

In our system, collaboration between the edge and cloud servers is crucial for optimizing performance and minimizing latency. The edge server, implemented on the ESP32-CAM, handles initial image capture and processing. It performs basic object classification and, when necessary, transmits results to the cloud server for detailed verification. The cloud server, using the computationally intensive YOLOv8 model, refines recognition and sends the final decision back to the edge server for action (e.g., triggering alerts, activating lights, or recording data). For example, in a security system, the edge server quickly detects movement and identifies a person in the video feed. The image is sent to the cloud for verification, where the YOLOv8 model confirms the identity and determines whether the individual poses a threat. This collaborative structure ensures responsiveness by leveraging the edge for speed and the cloud for accuracy. In YOLOv8, a confidence score represents the model’s certainty that an object exists within a bounding box. For instance, a detected person with a confidence score of 0.80 indicates an 80% likelihood of accurate identification. Scores below a predefined threshold are considered false positives and ignored. The system’s decision-making process balances real-time responsiveness with high recognition accuracy, leveraging the strengths of both edge and cloud computing.

**Figure 20:**
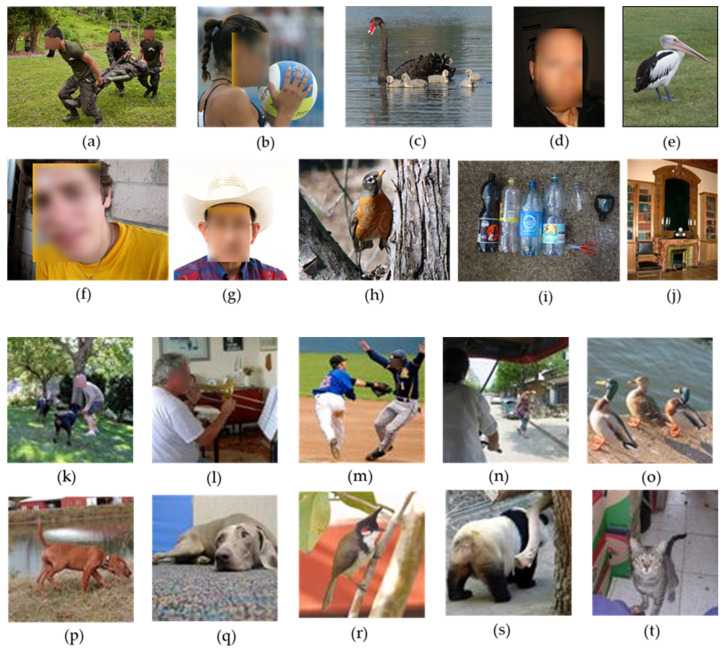
Samples of validation images: person (**a**,**b**,**d**,**f**,**g**,**k**,**l**,**m**,**n**), non-person (**c**,**e**,**h**,**i**,**j**,**o**,**p**,**q**,**r**,**s**,**t**).

In this paper, the threshold for the edge recognition is set at 0.5. If the confidence score is below 0.5, the result is classified as “non-person”, and the image is ignored. Conversely, if the confidence score is 0.5 or higher, the result is classified as “person”, and the image is sent to the cloud for further verification. We conducted 200 trials to validate the system’s performance. Selected sample images from ImageNet dataset, used for validation, are shown in Figure 20. The corresponding results, including confidence scores and correctness for both edge and cloud, are summarized in Table 4 and Table 5. For example:

1. Case (a): The edge recognition confidence score is 0.7, exceeding the threshold of 0.5. The image is classified as “person” and sent to the cloud, where YOLO assigns a confidence sco.re of 0.86, successfully identifying the person.
2. Case (c): The edge confidence score is 0.71, and the image is preliminarily classified as “person”. However, the cloud validation determines there is no person in the image, indicating a misclassification at the edge.
3. Case (h): The edge confidence score is 0.23, below the 0.5 threshold. The image is classified as “non-person” and ignored without being sent to the cloud.
4. Case (k): The edge recognition confidence score is 0.69, exceeding the threshold of 0.5. The image is classified as “person” and sent to the cloud, where YOLO assigns a confidence sco.re of 0.83, successfully identifying the person.
5. Case (o): The edge confidence score is 0.84, and the image is preliminarily classified as “person”. However, the test image contains no person. The misleading can be validated by the cloud.
6. Case (t): The edge confidence score is 0.64, exceeding the threshold. The image is classified as “person”. The misleading can be validated by the cloud, since the cloud confidence score is 0.31, less than the threshold 0.80.

Most misrecognitions happen at the edge due to the ESP32-CAM’s limited computational power and the use of a lightweight MobileNet model. This results in a correctness ratio of 0.76. The cloud server, equipped with a more complex YOLOv8 model and higher computational resources, achieves a much higher correctness ratio of 0.98. Since only images with high confidence are sent to the cloud for verification, errors made at the edge dominate the overall system performance, resulting in a combined accuracy of approximately 0.75.

## 4. Discussion

To assess the impact of different MQTT broker distances on latency, this study created a cloud environment using a Python program for publishing and subscribing to messages. Tests were conducted for Options 1 to 3 with three different MQTT brokers, involving the transmission of both text messages and images. The results are shown in Table 6. When only sending text messages, the local MQTT broker had a transmission time of approximately 2 ms, representing the basic response time for MQTT publishing and subscribe operations. For the domestic mqttgo.io broker, the time was 117 ms, while for the international mqtt.eclipseprojects.io broker, the time was 450 ms. These results indicate that server distance significantly impacts latency: the domestic server’s response time is 58.5 times that of the local server, and the international server’s is 3.85 times that of the domestic server. Such network latency can affect the immediacy of end devices relying solely on cloud processing. However, pre-processing through an edge server can effectively mitigate latency caused by network distance.

Reducing image size improves both the local processing speed of the ESP32-CAM and network transmission efficiency by minimizing the time required for image acquisition, processing, and transfer. Smaller images contain fewer pixels, reducing data size and thereby lowering bandwidth consumption. This directly impacts MQTT transfer time, as a smaller payload results in faster data transmission across the network.

In larger images, transmission delays increase, particularly in remote network scenarios where bandwidth limitations and congestion further slowdown data transfer. Additionally, the round-trip time which includes image capture, transmission, cloud-based recognition, and response delivery becomes significantly longer. A delayed response may cause the object of interest to move out of the field of view before an action can be taken.

In Option 1, every image is transmitted to the cloud, leading to higher bandwidth usage and increased latency. In contrast, Options 2 and 3 improve performance by executing initial recognition locally before determining whether to transmit the image to the cloud. This ensures that alerts or actions can be taken immediately, even before cloud verification, reducing response time in dynamic environments.

Experimental results show that, when sending images to a domestic broker, the average transmission time is 930 ms longer compared to a local broker within the same Wi-Fi network. For an international broker, this delay increases by 3192 ms, shown by Table 7. Referring to Table 1 and Table 2, at 640 × 480 resolution, the absolute time differences between brokers decrease, while the relative differences increase. This indicates that, while all brokers experience delays, the proportional impact of latency grows with increased transmission distance, reinforcing the benefits of edge-side processing.

Table 8 compares MQTT times across different brokers and image upload options, demonstrating that reducing image size from 640 × 480 to 320 × 240 nearly halves MQTT transmission time. This significantly enhances system performance by reducing network congestion, improving responsiveness, and optimizing bandwidth usage, making the edge–cloud architecture more practical and scalable.

**Figure 21:**
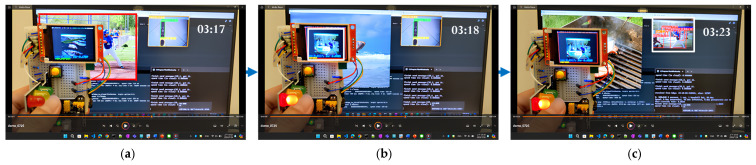
Snap shots of object recognition under domestic broker and Option 3: (**a**) a person is detected, (**b**) object recognition by the edge, (**c**) object recognition by the cloud.

Figure 21 illustrates the sequential results in the proposed edge–cloud object detection system. In each diagram of Figure 20, the timestamp is displayed in the top-right corner. At 03:17, a person is detected within the monitored area. The ESP32-CAM captures an image, processes it for recognition, and updates the result on the TFT-LCD, all within less than 1 s. Then, it takes approximately 5 s for the cloud to receive the image, perform recognition, and send the results back to the edge device. Experimental results show that, when connected to a domestic broker and using Option 3, “sending images only when a person is detected”, the timing diagram demonstrates that pre-processing enhances real-time performance, as reflected in the timing sequence from the video.

This study primarily focuses on evaluating MQTT transmission time and image sizing as key performance metrics for the proposed edge–cloud system. However, we recognize that a more comprehensive evaluation would include other important factors such as real-time processing capability and power consumption. Real-time processing capability is critical in applications that require immediate responses, while power consumption is an essential metric for battery-powered IoT devices. Future work will expand on this evaluation by measuring real-time processing times under various conditions and conducting a detailed analysis of power consumption to better understand the trade-offs involved in deploying the system in real-world scenarios. This study focuses on the implementation and evaluation of a single edge node system. However, scaling up the system to coordinate multiple edge nodes introduces additional challenges and opportunities. For example, efficient communication protocols, distributed workload balancing, and conflict resolution strategies would be critical in multi-node scenarios. Future work will explore the integration of coordination mechanisms, such as MQTT hierarchical topics or distributed edge–cloud frameworks, to support scalability in larger IoT networks. Additionally, testing the impact of multi-node coordination on latency, bandwidth usage, and power efficiency will be a focus of subsequent studies.

The ESP32-CAM is a resource-constrained device with limited processing power and memory, which impacts its ability to run complex deep learning models. The dual-core 240 MHz processor and 520 KB SRAM (with 4 MB PSRAM) constrain model complexity, inference speed, and memory usage. Instead of infeasible large models, we use MobileNet, which employs depthwise separable convolutions to reduce computation while maintaining accuracy. Due to memory constraints, input images are resized to 96 × 96 pixels, and batch processing is avoided to prevent instability. The hybrid edge–cloud approach enables quick local inference, while cloud-based YOLOv8 verification ensures higher accuracy without overloading the ESP32. This balance enhances real-time responsiveness, reduces network congestion, and improves system scalability. We have incorporated this discussion into the revised manuscript. Please let us know if further elaboration is needed.

## 5. Conclusions

This study demonstrates the effectiveness of integrating edge and cloud computing in an IoT-based image recognition system, using the ESP32-CAM as both an edge device and server. Through the deployment of a pre-trained MobileNet v1 model on the edge and the YOLOv8 model in the cloud, we evaluated a system that performs preliminary object recognition locally and verification in the cloud. The edge device communicates with the cloud via MQTT, ensuring efficient data exchange. Our experimental results show that edge-side pre-recognition and pre-processing significantly improve system performance, particularly when using a 320 × 240 image resolution. This approach reduces the workload on the cloud server, enhances overall computational efficiency, and optimizes system stability and response speed. Additionally, by transmitting only the portions of the image that change to the cloud, particularly in scenarios where objects (like people) are continuously present, the system saves network bandwidth and cloud storage space.

This work also highlights that developing edge server capabilities alongside cloud technologies creates a complementary architecture. This reduces latency and ensures real-time actions can be triggered, even when network delays exist, making the system suitable for time-sensitive applications like security monitoring. In summary, this ESP32-based edge computing framework illustrates the potential for scalable, AI-enhanced IoT solutions. By integrating edge and cloud computing, it addresses critical IoT challenges in latency and cloud dependency, opening possibilities for future smart applications.

## Figures and Tables

**Table 1:** Average loop time for different options across various brokers (640 × 480).

Avg. Loop Time (ms) Mean ± SD	Broker 1 Mosquitto	Broker 2 MQTTGO	Broker 3 Eclipse
Option 1	1047 ± 325	2702 ± 785	6862 ± 1682
Option 2	860 ± 18	995 ± 22	1396 ± 114
Option 3	1006 ± 193	2025 ± 1396	3577 ± 2898

**Table 2:** Average MQTT transmission time for different options across various brokers (640 × 480).

MQTT Time (ms) Mean ± SD	Broker 1 Mosquitto	Broker 2 MQTTGO	Broker 3 Eclipse
Option 1	449 ± 307	2112 ± 767	6265 ± 1664
Option 2	17 ± 6	182 ± 28	584 ± 110
Option 3	164 ± 193	1182 ± 1388	2734 ± 2906

**Table 3:** Average loop time for Eclipse broker at different times (640 × 480).

Avg. Loop Time (ms) Mean ± SD	Morning	Evening	Night
Option 1	5726±1877	5514 ± 1546	5503 ± 1413
Option 2	863 ± 96	837 ± 87	811 ± 84
Option 3	2808 ± 2973	2271 ± 2467	2053 ± 2497

**Table 4:** Validation results for the sample images.

Case	Image with Person	Confidence (Edge)	Correctness (Edge)	Confidence (Cloud)	Correctness (Cloud)
(a)	Yes	0.70	Yes	0.86	Yes
(b)	Yes	0.52	Yes	0.80	Yes
(c)	No	0.71	No	0.00	Yes
(d)	Yes	0.68	Yes	0.89	Yes
(e)	No	0.56	No	0.00	Yes
(f)	Yes	0.74	Yes	0.93	Yes
(g)	Yes	0.78	Yes	0.83	Yes
(h)	No	0.23	Yes	n/a	n/a
(i)	No	0.40	Yes	n/a	n/a
(j)	No	0.16	Yes	n/a	n/a
(k)	Yes	0.69	Yes	0.83	Yes
(l)	Yes	0.71	Yes	0.92	Yes
(m)	Yes	0.74	Yes	0.89	Yes
(n)	Yes	0.50	Yes	0.92	Yes
(o)	No	0.84	No	0	Yes
(p)	No	0.73	No	0	Yes
(q)	No	0.55	No	0	Yes
(r)	No	0.33	Yes	n/a	n/a
(s)	No	0.15	Yes	n/a	n/a
(t)	No	0.64	No	0.31	Yes

**Table 5:** Confusion matrix for the validation results.

		Actual	
Predicted		person	non-person
	person	9	1
	non-person	0	10

**Table 6:** Response time via different brokers.

Response Time (ms) Mean ± SD	Message	Image
Broker 1	2±1	10±8
Broker 2	117 ± 47	291±79
Broker 3	450 ± 64	1039 ± 358

**Table 7:** Average loop time for different options across various brokers (320 × 240).

Avg. Loop Time (ms) Mean *±* SD	Broker 1 Mosquitto	Broker 2 MQTTGO	Broker 3 Eclipse
Option 1	550 *±* 175	1480 *±* 329	3742 *±* 692
Option 2	459 *±* 80	640 *±* 158	1189 *±* 155
Option 3	613 *±* 214	1037 *±* 671	1996 *±* 1730

**Table 8:** Average MQTT transmission time for different options across various brokers (320 × 240).

MQTT Time (ms) Mean *±* SD	Broker 1 Mosquitto	Broker 2 MQTTGO	Broker 3 Eclipse
Option 1	336 *±* 119	1258 *±* 275	3522 *±* 665
Option 2	29 *±* 19	191 *±* 60	762 *±* 123
Option 3	75 *±* 198	278 *±* 650	1542 *±* 1725

## Data Availability

The original contributions presented in the study are included in the article, further inquiries can be directed to the corresponding author.
